# Changes in Functional Connectivity Following Treatment With Emotion Regulation Therapy

**DOI:** 10.3389/fnbeh.2019.00010

**Published:** 2019-02-04

**Authors:** Matthew A. Scult, David M. Fresco, Faith M. Gunning, Conor Liston, Saren H. Seeley, Emmanuel García, Douglas S. Mennin

**Affiliations:** ^1^Department of Psychiatry, Weill Cornell Medicine, New York, NY, United States; ^2^Department of Psychology & Neuroscience, Duke University, Durham, NC, United States; ^3^Department of Psychological Science, Kent State University, Kent, OH, United States; ^4^Department of Psychiatry, School of Medicine, Case Western Reserve University, Cleveland, OH, United States; ^5^Brain and Mind Research Institute, Weill Cornell Medicine, New York, NY, United States; ^6^Department of Psychology, University of Arizona, Tucson, AZ, United States; ^7^The Graduate Center, City College of New York, City University of New York (CUNY), New York, NY, United States; ^8^Hunter College, City University of New York, New York, NY, United States; ^9^Department of Counseling and Clinical Psychology, Teachers College, Columbia University, New York, NY, United States

**Keywords:** generalized anxiety disorder, major depressive disorder, worry, decentering, reappraisal, emotion regulation, resting state functional connectivity

## Abstract

Emotion regulation therapy (ERT) is an efficacious treatment for distress disorders (i.e., depression and anxiety), predicated on a conceptual model wherein difficult to treat distress arises from intense emotionality (e.g., neuroticism, dispositional negativity) and is prolonged by negative self-referentiality (e.g., worry, rumination). Individuals with distress disorders exhibit disruptions in two corresponding brain networks including the salience network (SN) reflecting emotion/motivation and the default mode network (DMN) reflecting self-referentiality. Using resting-state functional connectivity (rsFC) analyses, seeded with primary regions in each of these networks, we investigated whether ERT was associated with theoretically consistent changes across nodes of these networks and whether these changes related to improvements in clinical outcomes. This study examined 21 generalized anxiety disorder (GAD) patients [with and without major depressive disorder (MDD)] drawn from a larger intervention trial (Renna et al., 2018a), who completed resting state fMRI scans before and after receiving 16 sessions of ERT. We utilized seed-based connectivity analysis with seeds in the posterior cingulate cortex (PCC), right anterior insula, and right posterior insula, to investigate whether ERT was associated with changes in connectivity of nodes of the DMN and SN networks to regions across the brain. Findings revealed statistically significant treatment linked changes in both the DMN and SN network nodes, and these changes were associated with clinical improvement corresponding to medium effect sizes. The results are discussed in light of a nuanced understanding of the role of connectivity changes in GAD and MDD, and begin to provide neural network support for the hypothesized treatment model predicated by ERT.

## Introduction

Major depressive disorder (MDD) and generalized anxiety disorder (GAD) are two prevalent disorders with lifetime prevalence estimates ranging from 17 to 41% for MDD and 6% to 14% for GAD (Kessler et al., 2005; Moffitt et al., 2010). These conditions are also highly comorbid with one another (Kessler et al., 2003) which may account for a sub-optimal treatment response with otherwise efficacious treatments (Farabaugh et al., 2010, 2012). Given these high rates of diagnostic comorbidity and shared surface level clinical features, newer systems of nosology place MDD and GAD in a shared group that is commonly called the “Distress Disorders” (Watson, 2005). In addition, transdiagnostic approaches (e.g., Mennin et al., 2013; Mennin and Fresco, 2014; Barlow et al., 2017) have sought to identify common underlying disorder processes that cut across classification systems predicated primarily on symptom presentation (e.g., Nolen-Hoeksema et al., 2008; Watkins, 2008).

One candidate transdiagnostic feature common to distress disorders, especially MDD and GAD, is negative self-referentiality (e.g., worry, depressive rumination) which often takes the form of repetitive or perseverative reactive cognitive processes (Mennin and Fresco, 2013; Olatunji et al., 2013; Ottaviani et al., 2016). Negative self-referentiality characterizes the mental activity of individuals when they experience a discrepancy between their current emotional/motivational state and a representation of the future (i.e., planning), the past (i.e., failures/losses), or an idealized self (i.e., self-criticism). This self-conscious ability is normative and crucial for managing a world in which there is ambiguity and uncertainty (e.g., Mennin and Fresco, 2014). However, the tendency to engage in self-referential mental activity can become negatively reinforced *via* a perceived reduction in aversive emotions (Borkovec et al., 2004; Nolen-Hoeksema et al., 2008) especially during highly contrasting emotional states (i.e., positive emotions followed by negative emotions; Newman and Llera, 2011). Further, the propensity to engage in negative self-referentiality can result in considerable deficits in behavioral learning (Lissek, 2012; Whitmer and Gotlib, 2013).

Increasingly, findings from basic and affective science are converging on the neurobehavioral underpinnings of normative and disordered self-referentiality and its association with disorders such as MDD and GAD. For instance, considerable evidence identifies aberrant or excessive neural activity particularly in the default mode network (DMN; Hamilton et al., 2012, 2013; Whitfield-Gabrieli and Ford, 2012; Chen and Etkin, 2013; Andreescu et al., 2014). Similarly, task-based studies examining trait levels of worry or depressive rumination (Paulus and Stein, 2010; Hamilton et al., 2011) or instructions to worry or ruminate (Cooney et al., 2010; Paulus and Stein, 2010; Ottaviani et al., 2016) demonstrate focal activations in nodes of the DMN.

Another important transdiagnostic feature that marks distress disorders is known variously as neuroticism (e.g., Barlow et al., 2014), negative affectivity (e.g., Watson et al., 1988) or dispositional negativity (e.g., Shackman et al., 2016). This construct reflects a tendency to experience frequent and intense negative emotions including anxiety, fear, irritability, anger, or sadness, in response to various sources of stress (Barlow et al., 2014). Shackman et al. (2016) proposed that dispositional negativity is a definable construct reflected at many neurobehavioral levels of analysis (e.g., neural, peripheral, etc.) and is found broadly in nature (e.g., humans, non-human primates, rodents, etc.). This negative emotionality is characterized by under- and over-activation of reward and safety/threat systems respectively, as well as their co-occurrence (i.e., motivational conflict; Higgins, 1997; Klenk et al., 2011; Scult et al., 2016). However, unlike healthy individuals, individuals with distress disorders may be relatively less effective in resolving these motivation states and conflicts. One possible reason is that salience in one or both of these motivational systems may increase levels of subjective intensity and corresponding distress (Shackman et al., 2016). Self-report indices of neuroticism clearly predict a more severe and protracted course for mood and anxiety disorders (e.g., Brown, 2007; Brown and Rosellini, 2011; Barlow et al., 2014). Further, whereas diagnostic comorbidity has long been viewed as a predictor of an inferior treatment response (e.g., Mineka et al., 1998), high levels of neuroticism may contribute to the underperformance of otherwise efficacious treatments (e.g., Brown, 2007; Olatunji et al., 2010; Brown and Rosellini, 2011).

The salience network (SN; e.g., Craig, 2009; Menon, 2015) is involved in orienting attention to external and internal stimuli (Menon and Uddin, 2010), and facilitates the integration of sensory, emotional, and cognitive information in service of optimal communication, social behavior, and self-awareness (Menon, 2015). The insula is a central node which helps evaluate the impact of stimuli on the body (Paulus and Stein, 2006), including generation and regulation of affective responses and detection of emotionally salient stimuli (Paulus and Stein, 2010). Most research findings implicate the right anterior insula (e.g., Critchley et al., 2004) but increasingly, evidence also indicates a relevant role for the posterior insula in emotional processing as well (Kuehn et al., 2016). Negative self-referentiality including worry, may in fact exaggerate arousal (positive or negative; Pollatos et al., 2009; Paulus and Stein, 2010). Paulus and Stein (2010) posit that individuals with anxiety and depression exhibit a propensity to negatively interpret interoceptive afferents, resulting in increased sympathetic arousal, and in turn, increased escape or avoidance behaviors.

When examined *via* functional neuroimaging, patients with GAD and MDD frequently exhibit SN abnormalities (Etkin et al., 2009; Dutta et al., 2014; Kaiser et al., 2015). For instance, compared to healthy individuals, depressed patients show reduced connectivity between anterior insula and other nodes of the SN (Manoliu et al., 2014; Yuen et al., 2014). In task-based studies, MDD and GAD patients consistently show hyperactivity of the anterior insula often accompanied by increased connectivity with nodes of DN including the posterior cingulate cortex (PCC; e.g., Paulus and Stein, 2010; Hamilton et al., 2013; Yuen et al., 2014). Similarly, a recent study by Kaiser et al. (2015) found that in comparison to healthy control participants, patients with MDD evidenced increased connectivity of the MPFC to the insula and the strength of this connectivity was predictive of depression severity.

The frontoparietal control network (FPCN), with nodes in the dorsolateral prefrontal cortex (DLPFC) and posterior parietal cortex (PPC) is involved in “top-down control,” monitoring attention, and regulating sensory, and internal networks according to current task goals (Cole et al., 2014). MDD patients often demonstrate within-network hypoconnectivity in FPCN, and hypoconnectivity between the FPCN and the DMN (Mulders et al., 2015). Similarly, in MDD, hypoconnectivity between the FPCN and the dorsal attention network [DAN; underlying volitional deployment of attention toward stimuli and externally-directed cognitions (Corbetta et al., 2008)] may increase depressive rumination and decrease ability to attend to present-moment external stimuli, and thus loss of potential for corrective information for positive reappraisal or access to reward (Schooler et al., 2011). Dysregulation of FPCN may also underlie inefficiency in adaptive switching between task-relevant and irrelevant cognitions and behaviors, as well as deficits in top-down regulation of SN, which is hyperactive and hyper-connected in PTSD and GAD (Rabinak et al., 2011; Sripada et al., 2012; Sylvester et al., 2012; Wang et al., 2016; Akiki et al., 2017). In summary, the distress disorders, especially GAD and MDD, are prevalent and often comorbid conditions at both a diagnostic and symptom level of analysis. When looking beyond surface characteristics, distress disorders exhibit excessive negative self-referentiality along with dispositional negative emotionality. These psychological characteristics are consistent with general hyperconnectivity within the DMN network, hypoconnectivity within the SN network and FPCN, and hypoconnectivity between the FPCN and DMN and DAN (Schooler et al., 2011; Mulders et al., 2015; Williams, 2016). Efforts focused on correcting these circuit-level abnormalities through targeted psychological and pharmacological interventions may result in a more efficacious treatment response.

Using this formulation of distress disorders as a conceptual model, Mennin and Fresco developed emotion regulation therapy (ERT), a theoretically-derived, mechanism focused treatment that integrates findings from affect science with principles from cognitive behavioral therapy (i.e., CBT; see Mennin et al., 2013) to target and normalize these neurobehavioral deficits (Fresco et al., 2013; Mennin and Fresco, 2015; Mennin et al., 2018; Renna et al., 2018b). ERT targets three hypothesized mechanisms: (1) motivational mechanisms, the functional purpose and inclinations of emotional response tendencies; (2) regulatory mechanisms, the ability to alter emotional responses both at less elaborative/attentional levels and more verbally elaborative and effortful levels including the ability to decenter (i.e., the meta-cognitive ability to observe items that arise in the mind with distance and perspective; present sample; Fresco et al., 2007; Bernstein et al., 2015) and reappraise (i.e., reinterpreting the meaning to change emotional trajectory; Ochsner and Gross, 2005); and (3) contextual learning, the use of flexible and adaptive behavioral repertoires, Using a motivational framework (i.e., identifying reward- and risk-based impulses), ERT instructs patients to engage in mindful emotion regulation skills to counteract negative self-referential processing (e.g., worry, rumination, and self-criticism) in service of pursuing intrinsically rewarding and goal-directed actions in their lives.

Three recently published trials of ERT attest to its efficacy in treating GAD and MDD (Mennin et al., 2015, 2018; Renna et al., 2018a). Following promising results from an initial open trial (Mennin et al., 2015), Mennin et al. (2018) found that GAD patients (with and without MDD) treated with 20 sessions of ERT vs. an attentional control intervention) evidenced statistically and clinically meaningful improvement on clinical indicators of GAD and MDD, worry, rumination, comorbid disorder severity, functional impairment, quality of life, as well as hypothesized mechanisms reflecting mindful attentional, metacognitive, and overall emotion regulation. The gains were maintained in post-treatment assessments 3- and 9-months following the end of treatment. In a secondary analysis of these trial data, Renna et al. (2018b) examined ERT-linked changes in behavioral tasks of flexible and sustained attention. Findings indicated that improvements in a specific form of attentional flexibility, conflict adaptation, predicted increases in mindful observing abilities whereas gains in sustained attention were related to mindful non-reactivity, clinical improvement, and decreased functional impairment.

Building on these encouraging efficacy findings, Renna et al. (2018a) utilized a 16-session format of ERT in an open trial design with an ethnically diverse sample of young adults. This trial, which is the parent study for the current study, reported impressive and durable efficacy in reducing worry, rumination, self-reported and clinician rated GAD and MDD severity, and social disability, while increasing quality of life, attentional flexibility, decentering/distancing, reappraisal, and trait mindfulness. In an initial secondary analysis of these trial data, we reported that baseline patterns of resting state functional connectivity (rsFC) within the DMN and SN predicted clinical response to ERT (Fresco et al., 2017). Specifically, higher baseline insula connectivity with parietal cortex, and aMPFC connectivity with precuneus and occipital cortex were associated with decreases in worry. Higher baseline PCC connectivity with the rostral ACC, and insula connectivity with lateral occipital cortex, central opercular cortex and dMPFC was associated with increases in decentering, while aMPFC connectivity with occipital pole was associated with decreases in decentering. Findings from this study implicated disruptions in the default and SNs as promising targets of treatment for GAD with and without co-occurring MDD but did not test how these networks might change as a result of treatment with ERT.

Beyond ERT, recent trials utilizing forms of mindfulness meditation have examined patterns of treatment linked rsFC change in their respective samples. In particular, Creswell et al. (2016) randomized subjectively-stressed unemployed adults to a 3-day intensive program of either mindfulness meditation, modeled after the mindfulness-based stress reduction curriculum (Kabat-Zinn, 2009), or a well equated relaxation curriculum. Participants completed a resting state scan before and after the intensive intervention. Seed-based change in functional connectivity using a seed in the PCC revealed that the mindfulness intervention, but not the relaxation intervention, was associated with increased connectivity between the PCC and left DLPFC. Comparable findings were reported by King et al. (2016) who randomized combat veterans with post-traumatic stress disorder to either 16 weeks of mindfulness-based exposure therapy (MBET), which was derived from mindfulness based cognitive therapy (Teasdale et al., 2000) and prolonged exposure therapy (Foa et al., 2007) or to a present-centered group therapy (PCGT; (Schnurr et al., 2003), a well equated comparator frequently used in PTSD trials. Consistent with Creswell et al. (2016), the PCC seed revealed that MBET but not PCGT was associated with the strength of functional connectivity between the left DLPFC, the right DLPFC, and the dorsal anterior cingulate cortex (dACC). Further, the strength of activation in the PCC-left DLPFC at post treatment was correlated with post-treatment PTSD avoidance symptoms (*r* = 0.623) and hyperarousal symptoms (*r* = 0.675) in patients receiving MBET but not PCGT. These findings combined with results from meta-analysis showing that individuals with depression tend to have decreased connectivity between PCC and DLPFC nodes compared to healthy controls (Mulders et al., 2015) raises the possibility that interventions for depression that include mindfulness meditation exercises, such as ERT, may lead to clinical improvement in part by increasing PCC-DLPFC connectivity.

The present study is drawn from a larger intervention trial (Renna et al., 2018a) and the baseline rsFC prediction study from the subset of the sample (Fresco et al., 2017). Findings from aforementioned trials with mindfulness interventions demonstrated changes in intrinsic functional connectivity in the DMN. Given these findings and our own baseline prediction findings, we sought to examine whether ERT would demonstrate similar patterns of rsFC changes in DMN and SN. Using seed-based connectivity analysis with seeds in the PCC, right anterior insula, and right posterior insula, we sought to identify patterns of ERT-linked rsFC changes of nodes within these networks across the brain and whether these changes would be associated with clinical improvement and ERT model related mechanism variables (e.g., attention control, decentering, and cognitive reappraisal) as well as reductions in MDD and GAD severity. Specifically, we hypothesized that ERT would be associated with decreased connectivity of nodes within the DMN, and that these changes would in turn be associated with decreased rumination. Increased connectivity of nodes within the SN would be expected to be associated with decreased depression and anxiety severity. Increased connectivity between nodes of the DMN and nodes of the FPCN would be expected to be associated with decreased depression and anxiety severity and improvements in attentional and metacognitive regulation (Mulders et al., 2015; Williams, 2016).

## Materials and Methods

### Participants

Participants were 25 treatment-seeking young adults, a subsample of the 31 patients treated in Renna et al. (2018a) who were drawn from an undergraduate and graduate student population in a large urban commuter-based university. Participants completed 16 weeks of ERT (Mennin and Fresco, 2014) and completed fMRI scans before and after treatment, with an average length of time between treatment and scan of less than 2 weeks. Participants were recruited through direct referrals from an on-campus counseling center, fliers posted throughout campus, e-mail announcements sent to the entire student body, and through research staff handing out business cards to students on campus. Four patients were excluded for technical issues that arose during MRI acquisition that resulted in unusable MRI data. The final sample had a mean age of 21.8 years old (*SD* = 2.6, range 18–27). Sixteen participants were female (76.2%). Seven participants identified as Hispanic and 14 as non-Hispanic. Additionally, participants identified primarily as White (8), followed by Asian (5), Other/mixed race (7), and Black (1).

### Inclusion/Exclusion Criteria

The main eligibility criterion was the presence of a primary or secondary GAD diagnosis. In the current study, 16 patients had a primary diagnosis of GAD (primacy based on symptom severity). Sixteen patients also met criteria for MDD; 14 patients met criteria for at least one additional anxiety disorder diagnosis. Other diagnoses included social anxiety disorder (*n* = 10), panic disorder (*n* = 6), specific phobia (*n* = 4), obsessive compulsive disorder (*n* = 3), post-traumatic stress disorder (*n* = 1). Participants were required to be stabilized on any psychotropic medications for a period of at least 3 months prior to the start of treatment (*n* = 1 receiving antidepressant medication) and could not be enrolled in any other form of psychological treatment during the acute phase of ERT (16 weeks). Participants were not taking any other medications at the time. Finally, participants had to be free of active suicidal ideation/intent, psychosis, bipolar I disorder, primary anorexia or bulimia nervosa, somatoform disorders, or substance and alcohol dependence. Given the use of fMRI assessment, other exclusionary criteria included standard MRI contraindications (e.g., ferromagnetic implants; head trauma with loss of consciousness; tattoos above the elbow; pregnancy).

### Diagnostic Assessment

Current and lifetime psychiatric disorders were assessed with the Structured Clinical Interview for DSM-IV (SCID; First et al., 2002). Graduate students and senior research assistants, extensively trained on the diagnostic assessment protocol administered this assessment. A principal investigator and an independent assessor, both of whom were blind to the participant’s diagnoses assigned at the intake interview, then confirmed participants’ diagnoses. Reliability was high, with kappa ratings ranging from 0.708 to 1.000, demonstrating good to excellent reliability. Reliability for diagnoses of GAD was 100%, whereas MDD was 87.10%. Independent assessors, who remained blind to treatment status of patients, assessed clinical improvement at mid-treatment, post-acute treatment, as well as 3-, and 9-months following the end of treatment.

### Treatment

ERT consists of 16-session individual weekly sessions completed within a 20-week span. The first half of the treatment (Phase I) emphasizes psychoeducation and cultivating mindful emotion regulation skills. Participants receive instruction in attention regulation (i.e., orienting, allowing) and meta-cognitive regulation (i.e., decentering, and cognitive reappraisal) skills. In particular, clients are instructed on how to better attend to emotional and motivational cues that arise in daily life so that these cues are noticed with greater acuity and closer to when they first arise. This cue detection is supported by a variety of meditation practices that improve attention and metacognitive capacities that patients are asked to practice daily. Briefer versions of these meditation practices are also introduced so that they can be utilized in both predicted and impromptu stressful situations as an alternative to negative self-referentiality and behavioral responses associated with escape or avoidance. The second half of treatment (Phase II) focuses on context engagement, which involves developing a proactive approach towards life with the goal of living more consistently with one’s values through the use of imaginal exposures and internal dialog tasks. Here, therapists direct patients in conducting in-session exposure exercises where patients envision a situation, goal, or outcome that they desire but is presently missing from their lives. This imaginal exposure serves to elucidate the motivational inclinations for reward and approaching a goal as well as the motivations associated with protecting one’s self from the threat associated with taking the action and/or costs associated with not succeeding. By giving voice to these motivational inclinations, patients learn to decenter from the intensity of these pulls and derive a behavioral response that reflects an optimal balance of risk and reward. More information regarding the structure and specific components of ERT are described elsewhere (see Fresco et al., 2013; Mennin and Fresco, 2014; Renna et al., 2017).

Clinicians consisted of seven doctoral students in clinical psychology who were trained to administer ERT and received 2 h of weekly supervision. The modal number of cases treated by each clinician was three (*M* = 2.75; range = 1–4). To establish adherence to the treatment protocol, all treatment sessions were audio recorded, and a team of research assistants, not involved in the administration of ERT or assessment of treatment effects, coded 40% of all cases, with 25% of these cases reviewed by a second coder to establish reliability. Reliability rates between the coders were 100%. Coders rated the accuracy of the frequency and skillfulness of actions taken by the study therapists. Overall, skillfulness ratings of the therapists coded were 98.4% (range = 95%–100%), while frequency of actions consistent with the treatment protocol was 91.2% (range = 71%–100%). The adherence ratings for this trial indicate that therapists uniformly delivered ERT with a high degree of adherence and fidelity. Examination of treatment effects associated with particular clinicians revealed equivalence for self-report and clinician- assessed clinical outcomes (*p*’s > 0.70) across the seven trial therapists.

Each diagnosis reaching clinical or subclinical thresholds was assigned a clinical severity rating (CSR) score from 0 to 8, based on criteria outlined in and adapted from the *Anxiety Disorders Interview Schedule for DSM-IV* (ADIS; Brown et al., 1994). Diagnostic criteria at the subclinical threshold for a given disorder are reflected by a CSR less than four. A CSR of four or above indicates that all criteria for a diagnosis were endorsed at the clinical threshold, with higher scores indicating greater severity. Interviewers were trained to assign these scores as per ADIS guidelines based on number and frequency of symptoms endorsed, while also taking into account related levels of distress and impairment attributed to the disorder symptomatology.

### Clinical Outcomes

Clinician assessed severity for GAD and MDD were determined by an independent assessor using the ADIS CSR rating for GAD and MDD. Details on assessment and training of these independent assessors and the deriving of these ratings are available in Renna et al. (2018a).

*The Penn State Worry Questionnaire* (PSWQ; Meyer et al., 1990) is a 16-item self-report measure of pathological worry with scores ranging from 16 to 80. Cronbach’s alpha in the current sample was good (*α* = 0.80).

The *Brooding subscale of Response Styles Questionnaire* (RS; Treynor et al., 2003; Armey et al., 2009) is a five-item measure of self-reported rumination free of depression symptom content. Internal consistency for the RS in the current study was moderate at 0.63.

The *Attentional Control Scale* (ACS; Derryberry and Reed, 2002) is a 20-item measure with two subscales that assess the degree to which an individual is able to shift and sustain/focus their attention. Higher scores indicate greater ability to control one’s attention. Internal consistency in the current study at pre-treatment was strong (*α* = 0.85 for entire scale, *α* = 0.80 for Focusing Attention, *α* = 0.73 for Shifting Attention).

*The Experiences Questionnaire-Decentering Subscale* (Decentering; Fresco et al., 2007) is an 11-item measure assessing the meta-cognitive strategy of decentering often defined as viewing oneself as separate from their emotional experience. Cronbach’s alpha in the current sample was good (*α* = 0.80).

*The* *Emotion Regulation Questionnaire—Reappraisal subscale* (ERQ-R; Gross and John, 2003) is a six-item measure of cognitive reappraisal that demonstrated strong internal consistency in the current study at pre-treatment (*α* = 0.86).

*The Mood and Anxiety Symptom Questionnaire-Short Form* (MASQ-SF; Clark and Watson, 1991) is a 62 item measure assessing anxiety and depression symptoms. The four factors derived from the MASQ represent: General Distress Anxiety (MASQ–GDA), Anxious Arousal (MASQ–AA), General Distress Depression (MASQ–GDD), and, Anhedonic Depression (MASQ–AD). Cronbach’s alpha for the MASQ subscales in the current study ranged from moderate to strong at pre-treatment (α’s = 0.61–0.91).

### Procedure

The Institutional Review Board of the college approved all aspects of the study. Participants provided written informed consent for all procedures at the outset of study. At the initial intake visit participants were assessed for current and lifetime psychiatric history *via* the SCID interview and also completed a battery of self-report questionnaires delivered in paper-and-pencil format. Prior to the start of treatment, participants completed an independent assessment with a different interviewer who re-assessed the diagnoses that were of clinical threshold at the initial intake. Finally, participants completed the fMRI scan. Following the first eight sessions (i.e., mid-treatment) and after 16 sessions (i.e., post-treatment), participants returned to the lab to complete another independent assessment and self-report questionnaire packet. They also completed another fMRI session post-treatment. Participants were compensated for all research related study visits.

### Analytic Plan

MRI Data Acquisition Imaging data were collected on a 3.0T Siemens Allegra head-dedicated MRI scanner with a standard quadrature head coil at the NYU Center for Brain Imaging in New York, NY, USA. Scan sessions lasted 90 min during which participants completed a resting state fMRI scan, and an anatomical scan, and three task-based scans (not examined in the current study). The resting state scan was always acquired prior to the task-based scans. During the 6-min resting-state sequence, participants were asked to keep their eyes open while a white crosshair was displayed on a black screen. The resting-state scan comprised 180 contiguous whole-brain functional volumes, acquired using a multi-echo echo planar imaging (EPI) sequence (repetition time = 2,000 ms; echo time = 30 ms; flip angle = 90°; 33 slices; matrix = 64 × 64; voxel size = 3 × 3 × 4 mm). High-resolution T1-weighted MPRAGE structural images (TR = 2,500 ms; TE = 3.93 ms, flip = 8°, 1 × 1 × 1 mm voxels) were acquired to facilitate localization and coregistration of functional data.

### MRI Data Preprocessing

MRI preprocessing was undertaken in AFNI (Cox, 1996) following the steps detailed in Power et al. (2017). To correct for subject movement, FD and DVARS were calculated before any other preprocessing steps were performed. Despiking was performed using AFNI’s 3dDespike for the entire volume. Slice time correction was performed using 3dTShift, shifting all signals to the time when the volume began to be collected, specifying interleaved acquisitions with an odd number of slices, and using the heptic Lagrange polynomial interpolation. The scanner was already steady-state at initial acquisition, so no volumes were skipped at the beginning of the scan. Realignment was conducted with 3dvolreg, using the first volume of a scan as the reference.

Registration of fMRI data to atlas space was conducted next. AFNIs @auto_tlrc command was used to register the first volume of the fMRI scan to each subject’s MP-RAGE, and all fMRI scans were registered to the first volume of the fMRI scan in the motion correction step. Registrations were then concatenated to a single transform, which was transformed into AFNIs TT_N27 atlas space and resampled to 3 mm isotropic voxels. All T1-weighted images underwent automated segmentation using FreeSurfer version 6.0, implemented with the recon-all command.

Time-series images for each participant were further processed to limit the influence of motion and other artifacts. Motion regressors were created using each subject’s six motion correction parameters (three rotation and three translation) and their first derivatives (Jo et al., 2013; Satterthwaite et al., 2013) yielding 12 motion regressors. White matter and cerebrospinal fluid nuisance regressors were created using CompCorr (Behzadi et al., 2007). Images were bandpass filtered to retain frequencies between 0.008 and 0.1 Hz, and volumes exceeding 0.25 mm frame-wise displacement or 1.55 standardized DVARS (Power et al., 2014; Nichols, 2017) were censored. Nuisance regression, bandpass filtering and censoring for each time series was performed in a single processing step using AFNI’s 3dTproject. One patient was excluded from subsequent analyses due to not passing QA procedures. Additionally, one subject’s baseline scan and another subject’s follow-up scan were excluded for not passing QA procedures, but each of their corresponding scans were included in the group-level rsFC analyses.

### Resting State Functional Connectivity (rsFC): Seed-Based Analyses

To investigate changes in connectivity of nodes within the DMN and SN, particular seeds within the DMN (PCC) and SN (Insula) were chosen. Specifically, ROIs were defined based on Fresco et al. (2017). For the PCC, a 2 mm sphere was created around the coordinates (−8, −56, 26). The right anterior insula and right posterior insula seeds (*K* = 2 clusters per hemisphere) were created by Kelly et al. (2012) and downloaded for the present study from the 1,000 Functional Connectomes Project^1^. For each seed, mean timeseries were extracted and used to create whole brain Z-transformed correlation maps for each participant. Group level analyses were conducted using AFNIs 3dLME (Chen et al., 2013) testing pre- to post-treatment change in rsFC. 3dLME was chosen to be able to account for missing data in repeated measures designs.

Correction for multiple comparisons was conducted using AFNI’s 3dClustSim (version 17.3.06) for cluster-size thresholding based on Monte Carlo simulation. An initial, uncorrected, statistical threshold of *p* < 0.01 with option NN1 (faces must touch) was chosen (Cox et al., 2017). Based on this threshold, the number of comparisons in our imaging volume and the smoothness of our imaging data, as measured by 3dFWHMx -acf, a minimum cluster size of nine voxels was required to have a corrected *p* ≤ 0.05 with 2-sided thresholding.

Significant clusters were saved as a mask and mean parameter estimates from the clusters were extracted from pre- and post-test scans using 3dROIstats to be entered into statistical models in IBM SPSS Statistics 24 (Chicago, IL, USA).

### Associations Between Change in Resting State Functional Connectivity With Clinical Variables

Time 2 rsFC and clinical variables were regressed onto their Time 1 counterparts and the unstandardized residual was saved as a new variable. We examined the zero order correlations among rsFC change indices with clinical change indices. Given the small sample size of the study, we elected to interpret correlations of at least a medium effect size (*r* > 0.30; Cohen, 1992) and made note of when these correlations also reached conventional probability values (*p* < 0.05).

## Results

### ERT Linked Clinical Improvement

Mean levels of clinical variables pre- and post-therapy are shown in Table 1. The results for the subsample of participants included in the current article are comparable to those found in the parent study (Renna et al., 2018a). Participants demonstrated a significant decrease in clinician assessed severity of GAD and MDD symptoms as well as in rumination and worry. Participants also demonstrated a significant increase in emotion regulation skills of attentional control (both shifting and focusing), decentering, and reappraisal. All clinical indicators exceeded conventions for large effect sizes (Hedges *g* > 0.80).

**Table 1:** Means and standard deviations of emotion regulation therapy (ERT) linked clinical outcomes.

	Pre-treatment	Post-treatment	*t*(df)	*p*	Hedge’s *g*
*GAD CSR*	5.8 (0.7)	3.4 (0.9)	10.0 (20)	<0.001	2.73
*MDD CSR*	4.4 (1.0)	2.3 (1.6)	5.1 (16)	<0.001	1.48
*Rumination*	14.8 (2.8)	10.2 (4.2)	4.5 (20)	<0.001	1.26
*Worry*	70.4 (6.4)	48.9 (12.6)	8.4 (20)	<0.001	2.08
*MASQ-GDA*	31.0 (5.8)	19.8 (5.1)	7.2 (20)	<0.001	1.97
*MASQ-GDD*	41.6 (8.2)	23.1 (9.7)	6.1 (20)	<0.001	1.98
*Attentional control*	44.5 (8.8)	51.6 (7.9)	4.0 (20)	0.001	0.82
*Reappraisal*	20.3 (7.6)	29.3 (8.0)	3.8 (20)	0.001	1.11
*Decentering*	24.9 (6.8)	38.2 (9.3)	5.4 (20)	<0.001	1.57

*Note: GAD, Generalized Anxiety Disorder; CSR, Clinician Severity Rating; MDD, Major Depressive Disorder; MASQ-GDA, Mood and Anxiety Symptom Questionnaire, General Distress Anxiety; MASQ-GDD, Mood and Anxiety Symptom Questionnaire, General Distress Depression*.

### ERT Linked Change in rsFC

**Figure 1:**
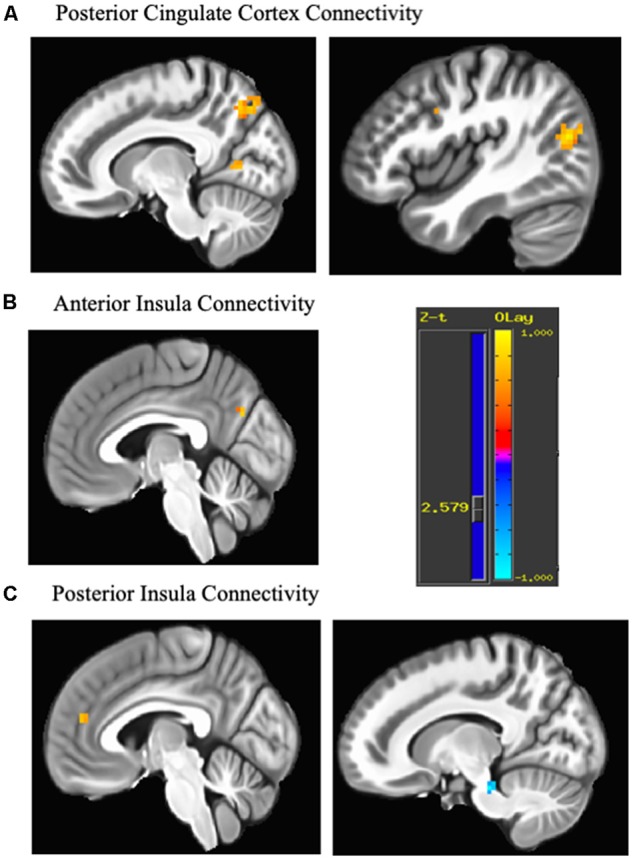
Change in connectivity associated with each seed. Regions demonstrating pre-post emotion regulation therapy (ERT) change in resting state functional connectivity (rsFC). **(A)** The posterior cingulate seed showed increased connectivity with middle occipital gyrus (43, −74, 17), precuneus (13, −68, 47), cuneus (16, −65, 11), precentral gyrus (−41, −5, 53; not shown) and premotor areas/dorsolateral prefrontal cortex (DLPFC; 40, 4, 35). **(B)** The anterior insula seed showed increased connectivity with the cuneus (−5, −71, 29). **(C)** The posterior insula seed increased connectivity with anteromedial PFC/dorsal anterior cingulate cortex (dACC; −5, 46, 17) and decreased connectivity with midbrain (13, −29, −25). Cluster are significant after cluster-based correction for multiple comparisons (>9 contiguous voxels). Yellow scale indicates positive z-scores, and blue scale indicates negative z-scores.

The posterior cingulate seed demonstrated increased connectivity from pre- to post-treatment with five cortical regions consisting of the middle occipital gyrus [Right Brodmann Area (BA) 19], precuneus (Right BA 7), cuneus (Right BA 17), precentral gyrus/motor cortex (Left BA 6) and premotor areas/DLPFC (Right BA 8/9). The anterior insula seed evidenced increased connectivity with precuneus (Left BA 18), while the posterior insula seed showed increased connectivity with anteromedial PFC/dACC (Left BA 32/10) and decreased connectivity with midbrain (Table 2 and Figure 1).

**Table 2:** Change in connectivity associated with each seed, listed by cluster size and MNI coordinates of peak voxel.

Seed	With region	BA	Cluster size	*x*	*y*	*z*	Max Z
**Post > Pre**
PCC	Middle Occipital Gyrus	19	52	43	−74	17	4.66
PCC	Precuneus	7	35	13	−68	47	3.77
PCC	Cuneus	17	22	16	−65	11	3.56
PCC	Precentral Gyrus	6	22	−41	−5	53	4.08
PCC	Pre-motor areas/DLPFC	8/9	20	40	4	35	3.65
raInsula	Cuneus	18	9	−5	−71	29	3.93
rpInsula	Anteromedial PFC/dACC	10/32	10	−5	46	17	4.13
**Pre > Post**
rpInsula	Midbrain	n/a	9	13	−29	−25	−4.24

*The top five significant clusters are presented for each seed. Note: BA, Brodmann Area; PCC, Posterior Cingulate Cortex; raInsula, Right Anterior Insula; rpInsula, Right Posterior Insula; DLPFC, Dorsolateral Prefrontal Cortex; dACC, Dorsal Anterior Cingulate Cortex*.

### Association of rsFC Change to Clinical Improvement

Table 3 displays zero order correlations between residual change in functional connectivity and clinical outcomes attributable to ERT. Few statistically significant associations were found between the residual change in extracted cluster values and residual change in clinical improvement or measures of emotion regulation. However, findings did reveal a pattern of correlations between rsFC change and clinical outcomes above the threshold for a medium effect size and/or probability values less than 0.05, that may achieve traditional statistical significance with a large sample. For instance, three of the clusters associated with the PCC seed evidenced moderately larger correlation coefficients. In particular, increases in functional connectivity between the PCC-Middle Occipital Gyrus cluster was positively correlated with ERT linked gains in attentional control, decentering, and cognitive reappraisal. Similarly, increases in functional connectivity between the PCC and Precentral Gyrus (Motor Strip) were negatively correlated with ERT linked reductions in GAD severity, anxiety and depression distress, and rumination, as well as gains in decentering and cognitive reappraisal. Increases in functional connectivity between the PCC and premotor areas/DLPFC were negatively correlated with reductions in MDD severity and positively correlated with gains attention control, decentering, and cognitive reappraisal. Finally, functional connectivity of the PCC with the cuneus was associated with ERT-linked gains in attentional control, whereas, PCC connectivity with the precuneus was associated with ERT-linked gains in reappraisal. On balance, rsFC clusters emerging from right anterior insula and right posterior insula seeds were not meaningfully correlated with ERT linked changes on clinical indicators.

**Table 3:** Association of ERT linked rsFC change to clinical improvement and model related mechanisms.

Resting state functional connectivity		Clinical indicators						Emotion regulation mechanisms			
		Δ GAD severity	ΔMDD severity	Δ Anxiety distress	Δ Depression distress	Δ Worry	Δ Rumination	Δ Focus	Δ Shifting attention	Δ Decentering	Δ Reappraisal
**PCC seed with**										
Middle Occipital Gyrus	*r*	−0.063	0.164	0.004	0.264	−0.218	−0.155	0.287	0.512*	**0.373**	**0.358**
	*p*	0.798	0.530	0.986	0.274	0.370	0.528	0.233	0.025	0.116	0.132
Precuneus	*r*	−0.215	−0.045	0.066	0.138	**0.336**	0.284	−0.092	0.025	0.116	**0.307**
	*p*	0.376	0.863	0.789	0.572	0.160	0.239	0.709	0.920	0.636	0.202
Cuneus	*r*	0.123	0.221	0.234	**0.353**	−0.110	−0.005	**0.339**	0.507*	0.163	0.213
	*p*	0.615	0.395	0.335	0.139	0.654	0.983	0.156	0.027	0.506	0.381
Precentral Gyrus (Motor)	*r*	**−0.419**	−0.148	−0.569*	**−0.395**	−0.226	**−0.318**	0.209	0.191	**0.355**	**0.380**
	*p*	0.074	0.570	0.011	0.094	0.352	0.184	0.391	0.433	0.136	0.108
Pre-Motor areas/DLPFC	*r*	0.101	**−0.417**	−0.194	**−0.367**	−0.049	−0.083	**0.369**	**0.336**	**0.320**	**0.403**
	*p*	0.681	0.096	0.427	0.122	0.842	0.736	0.120	0.160	0.182	0.087
**rpInsula seed with**										
amPFC/dACC	*r*	−0.144	0.246	−0.025	−0.152	−0.272	−0.027	−0.084	−0.231	0.060	−0.088
	*p*	0.557	0.341	0.920	0.533	0.260	0.911	0.733	0.341	0.808	0.720
Midbrain	*r*	−0.087	**−0.346**	0.070	0.018	−0.103	0.209	−0.046	0.120	−0.046	0.017
	*p*	0.724	0.173	0.777	0.943	0.676	0.391	0.851	0.624	0.850	0.945
**raInsula seed with**										
Precuneus	*r*	0.202	0.170	−0.030	0.138	−0.104	−0.052	−0.1290.50	−0.129275	−0.239	−0.260
	*p*	0.936	0.514	0.903	0.573	0.673	0.831	0.600829	0.600228	0.324	0.282

Note: PCC, Posterior Cingulate Cortex; raInsula, Right Anterior Insula; rpInsula, Right Posterior Insula; DLPFC, Dorsolateral Prefrontal Cortex; dACC, Dorsal Anterior Cingulate Cortex; Correlations in bold exceed conventions for a medium effect size (r > 0.30), *p < 0.05.

## Discussion

This study represents the first investigation of changes in rsFC following treatment with ERT, a theoretically-derived, mechanism focused treatment for distress disorders that was developed to target and normalize negative motivational salience and subsequent self-referential processes as reflected in hypothesized neurobehavioral deficits in the DMN and SN (i.e., hyperconnectivity within the DMN network, hypoconnectivity within the SN network and FPCN, and hypoconnectivity between the FPCN and DMN). In this study drawn from a larger intervention trial (Renna et al., 2018a), we utilized a seed-based connectivity analysis with seeds in the PCC, right anterior insula, and right posterior insula. Findings revealed changes in connectivity of nodes in the DMN and SN networks with other nodes in these networks and with other cortical regions post-therapy compared to pre-therapy. Five clusters derived from the PCC seed and three clusters derived from insula seeds were retained and examined in relation to ERT linked improvements in clinical indicators of GAD and MDD severity, worry, rumination, as well as mechanistic emotion regulation variables (e.g., focusing and shifting attention, decentering, cognitive reappraisal). Meaningful and theoretically consistent correlations emerged between PCC seeded clusters and clinical variables of moderately large effect size, but because of the relatively small sample size of the study, only a few achieved conventional thresholds of statistical significance.

Following treatment with ERT, the PCC seeds revealed increased connectivity with a region that includes pre-motor cortex and posterior DLPFC, findings consistent with two recent trials that utilized mindfulness-based interventions (Creswell et al., 2016; King et al., 2016). In these studies, increased connectivity between the PCC and DLPFC was associated with post-treatment PTSD symptoms (King et al., 2016) and reduced serum inflammatory markers (Creswell et al., 2016). Similarly, increased DLPFC function has also been associated with reappraisal (Ochsner et al., 2002; Scult et al., 2017b), and with decreased anxiety (Scult et al., 2017a). The present results also found a trend for this increase in PCC-pre-motor/DLPFC connectivity to parallel decreases in MDD severity and depression distress, and increases in attentional control and emotion regulation. These results fit with previous work showing a unique functional coupling of DLPFC and PCC in instances of cognitive control (Smith et al., 2016), suggesting that the ERT intervention may have enhanced cognitive control of emotional processing through increasing PCC-DLPFC coupling at rest. Increasing connectivity of other brain regions such as the medial PFC (Etkin et al., 2011) with the posterior insula may reflect the appraisal of emotional responses *via* more metacognitive processes that create an empathic distance from the emotion itself (similar to the empathy experienced for the distress of others; Lamm et al., 2011), and indeed this increased connectivity showed a trend for increasing decentering in the present results.

The increases in connectivity of the PCC with other regions of the DMN (e.g., precuneus) were contrary to hypothesis, given the well-documented patterns of hyperconnectivity within the DMN in depression (Kaiser et al., 2015) which are sometimes normalized with antidepressant medication (Posner et al., 2013). However, recent research suggests that a focus on overall DMN connectivity may be overly simplistic, and that instead, connectivity between anterior portions of the DMN may be positively correlated with anxiety and depression symptoms while connectivity between posterior nodes of the DMN may be negatively correlated with depression and anxiety symptoms (Coutinho et al., 2016). Our results of increasing connectivity of the PCC with other posterior regions both within and beyond the DMN (precuneus, cuneus, middle occipital gyrus) after ERT treatment, paralleling decreases in mood and anxiety symptoms, fit within this framework as further described below.

In particular, the present study found changes in connectivity of brain regions involved in shifting attention towards important situational cues. The PCC has been implicated in self-generated thought irrespective of whether attention is focused internally or externally, while middle occipital gyrus activity has been associated with externally directed attention (Benedek et al., 2016). Areas of the medial PFC overlapping with activations found in the present study showing increased connectivity with posterior insula, have been associated with positively valenced self-related processing (Johnson et al., 2009). Meanwhile, the precentral gyrus is involved in intentional motor activity (Kana et al., 2015). One potential interpretation of these patterns of activation is that these regions are implicated in agentic thoughts and actions, which stands in contrast to the experience of individuals with elevated anxiety and depression, who often overlook overt cues for reward and have difficulty accurately assessing environmental cues signaling danger (Renna et al., 2017). In healthy individuals, DMN and SN activity is linked with processing of internal and external cues that are related to situational awareness. For example, the middle occipital gyrus has been implicated in mentalizing or inferring the emotions of others (Atique et al., 2011; Schurz et al., 2014), while PCC activation has been associated with agentic control (Brewer and Garrison, 2014). One possible explanation, therefore, is that ERT may act by increasing the ability of individuals to accurately shift attention to cues in the environment *via* enhanced connectivity of regions related to perceptual processing and mentalizing (Ganis et al., 2004; Schurz et al., 2014), which in turn, leads to the alleviation of anxious and depressive symptoms.

An important guiding principle of ERT is the contention that refractory conditions such as distress disorders require intervention components that target attention and metacognitive capacities to produce a meaningful and durable treatment response (Fresco and Mennin, 2019). Several reported findings herein are potentially consistent with that premise. For instance, we conducted some *post hoc*, unplanned tests of dependent correlations (Steiger, 1980) comparing the strength of correlation with self-report measures of attention and metacognition to the extract clusters associated with ERT-linked neural change. Findings revealed that rsFC change in the cuneus, an area generally implicated in spatial attention (Simpson et al., 2011) especially when cues may convey threat or anger (Heesink et al., 2017), was more strongly associated with ERT-linked changes in shifting attention as compared with indicators of metacognitive change-decentering (*t* = 2.59, *p* = 0.02, Cohen’s *d* = 1.22) and reappraisal (*t* = 1.82, *p* = 0.08, Cohen’s *d* = 0.86). Conversely, rsFC change in the precuneus, a node of the DMN implicated in self-consciousness and self-related mental representations (e.g., Cavanna and Trimble, 2006) was more strongly correlated with ERT-linked gains in reappraisal as compared to gains in focused attention (*t* = 2.02, *p* = 0.04, Cohen’s *d* = 1.04) and shifting attention (*t* = 1.61, *p* = 0.12, Cohen’s *d* = 0.76). Finally, rsFC changes in the middle occipital gyrus, implicated with both attention (Benedek et al., 2016) and metacognition (Atique et al., 2011; Schurz et al., 2014) were similarly correlated with ERT-linked gains in attention, decentering, and reappraisal. Future research may wish to examine these areas for future seed-based analyses, ideally with a larger treatment sample.

There are several limitations of the present study. In particular, this study was preliminary and lacked a control group or treatment comparison, which raises caution in interpreting the findings. Future research, utilizing a randomized controlled trial design is the logical next step to determine what changes are uniquely related to ERT. Similarly, the study was conducted with a modest sample size and given the interest in investigating multiple nodes within the default mode and SN with several clinical variables of interest, larger samples will be needed in the future to robustly test the associations between these variables, as well as to assess moderating factors such as sex.

Future studies will help to test the reliability of the present results and further elucidate a mechanistic understanding of the impact of ERT therapy on psychological and neurobiological variables. Despite the aforementioned limitations, the present findings add a level of nuance to the growing literature on rsFC disruptions in GAD and MDD and highlight the potential impact of treatment on connectivity in these disorders.

## Data Availability

Datasets are available on request: the raw data supporting the conclusions of this manuscript will be made available by the authors, without undue reservation, to any qualified researcher.

## Ethics Statement

This study was approved by the Ethics Commitee of Hunter College Human Research Protection Program (HRPP). All participants in studies referenced gave full study consent prior to any research procedures.

## Author Contributions

MS, DF, FG, CL, SS, EG, and DM: substantial contributions to the conception or design of the work; the acquisition, analysis, or interpretation of data for the work, drafting the work or revising it critically for important intellectual content, final approval of the version to be published, agreement to be accountable for all aspects of the work in ensuring that questions related to the accuracy or integrity of any part of the work are appropriately investigated and resolved.

## Conflict of Interest Statement

The authors declare that the research was conducted in the absence of any commercial or financial relationships that could be construed as a potential conflict of interest.

## Footnotes

^1^http://fcon_1000.projects.nitrc.org
